# Integrative Taxonomy of Didymozoids Parasitizing *Thunnus obesus* (Scombridae) from Southwest Atlantic Ocean: A New Genus and Species

**DOI:** 10.3390/pathogens14040359

**Published:** 2025-04-07

**Authors:** Yuri C. Meneses, Marcia C. N. Justo, Ana Maria Moreira-Silva, Lorrayne S. S. de Brito, Alena M. Iñiguez, Simone C. Cohen

**Affiliations:** 1 Laboratory of Helminths Parasites of Fishes—LHPP, Oswaldo Cruz Institute, FIOCRUZ. Avenida Brasil, 4365, Rio de Janeiro 21040-360, RJ, Brazil; yuricosta202115@gmail.com (Y.C.M.); marciajusto@ioc.fiocruz.br (M.C.N.J.); ana.maria.moreira.silva@gmail.com (A.M.M.-S.); scohen@ioc.fiocruz.br (S.C.C.); 2 Pos Graduate Program in Biodiversity and Health, Oswaldo Cruz Institute, FIOCRUZ. Avenida Brasil, 4365, Rio de Janeiro 21040-360, RJ, Brazil; 3 Laboratory of Integrative Parasitology and Paleoparasitology—LPIP, Oswaldo Cruz Institute, FIOCRUZ. Avenida Brasil, 4365, Rio de Janeiro 21040-360, RJ, Brazil; lorraynebrito1992@gmail.com; 4 Pos Graduate Program in Parasite Biology, Oswaldo Cruz Institute, FIOCRUZ. Avenida Brasil, 4365, Rio de Janeiro 21040-360, RJ, Brazil

**Keywords:** Didymozoidae, *Platodidymocystis yamagutii* n. gen., n. sp., 28S rDNA, ITS2, South America coast, fish parasites

## Abstract

The global fauna of Didymozoidae infecting fishes is very diverse and includes 270 species. Integrative taxonomic studies in this group are rare, and genetic data are lacking for the molecular identification of species. In this study, a new genus and species, *Platodidymocystis yamagutii* n. gen., n. sp., is described based on morphological and genetic analyses. The new genus is allocated to Didymozoinae but differs from all other genera of this subfamily, mainly by the morphology of the testes and ovary. The genetic analysis and molecular phylogeny using 28S rDNA and ITS2 markers showed *P. yamagutii* n. gen., n. sp. in a unique cluster in monophyly and most closely related to *Platocystis vivipara* (Yamaguti, 1970), followed by *Didymocystis* spp. Additionally, *Didymosulcus philobranchiarca* (Yamaguti, 1970) was also characterized by integrative taxonomy and *Koellikerioides internogastricus* Yamaguti, 1970, *Didymocystis neothunni* (Yamaguti, 1970), and *P. vivipara* were analyzed by molecular taxonomy. This is the first integrative taxonomic study of Didymozoidae from the Atlantic coast and the first survey to provide novel genetic data for five species of Didymozoidae trematodes, contributing to the increment of the knowledge and expansion of the geographical distribution of didymozoid species parasites of Scombridae in the Southern Atlantic Ocean.

## 1. Introduction

The big eye tuna, *Thunnus obesus* (Lowe, 1839), is an important commercial epipelagic and mesopelagic species present in oceanic waters of the tropical and subtropical Atlantic, Indian, and Pacific Oceans but is absent in the Mediterranean Sea [1].

The high vagility and endothermy of tuna fishes require high metabolic energy, which is met through foraging on large quantities of food items, comprising crustaceans, fish, mollusks, and polychaetes, which serve as intermediate and paratenic hosts for helminth parasites [2], probably explaining the diversity of species of Didymozoidae in scombrid fishes.

Didymozoidae Monticelli, 1888 is a family of digeneans with several features different from other digeneans, constituting the most morphologically diverse group among trematodes [3]. The global fauna of Didymozoidae infecting fishes is diverse and includes 270 valid species belonging to 70 genera [4]. Members of this family are found parasitizing fishes, of which the most representative numbers are mainly in species of the Scombridae, among others, with a few exceptions from freshwater fishes. Most species of Didymozoidae were originally described in the Pacific Ocean, followed by the Indian Ocean and, to a lesser extent, in the Atlantic Ocean [3].

The first molecular approach to a didymozoid species in South America was the revalidation of *B. bennetti* [5], which was considered *incertae sedis* [6] and, later, the genus was considered as *genus inquirendum* [3]. 

An important genetic analysis of the Didymozoidae was carried out by Anderson [7], which provided subsidies to complement the identification by employing morphological analysis and including the family in the superfamily Hemiuroidea. Nevertheless, genetic studies of the group until now are scarce, mainly due to the complexity of the taxa [5,8,9]. Recent studies that contributed to genetic data on the family have demonstrated polyphyletic and paraphyletic genera in the topologies of the phylogenetic trees generated [9,10]. It is also possible to observe the availability of genetic data from specimens not curated by taxonomists specialized in Didymozoidae, without proper morphological characterization in integration with the generation of genetic data, which contributes to an unclear identification of the species [11]. Anyway, as proposed by these authors, the absence or scarcity of genetic sequences available for morphologically characterized didymozoids is clear, with several clades of species with only one or two representatives in the databases, many of these with a taxonomic identification at the level of genus, family, or tribe [10].

During a survey of the helminth parasites of *T. obesus* captured off the Southern Atlantic Ocean, several yellow capsules were observed under the dorsal skin and caudal fin. These parasites were allocated to Didymozoinae Monticelli, 1888 and are proposed as a new genus and species based on morphological and genetic analyses. In addition, new morphological data on *Didymosulcus philobranchiarca* are provided. This study contributes to the increment of the knowledge and expansion of the geographical distribution of didymozoid species parasites of Scombridae in the Southern Atlantic Ocean.

## 2. Materials and Methods

### 2.1. Fish Collection, Sampling, and Parasite Morphological Analysis

Forty-seven specimens of *Thunnus obesus*, 18 females [44–77 (57 ± 8.76) cm standard body length; 1.80–8.80 (4.09 ± 1.49) kg] and 29 males [41–73 (59 ± 7.92) cm standard body length; 1.80–6.80 (4.10 ± 1.30) kg], were obtained from fishermen in Cabo Frio, Rio de Janeiro, Brazil (22°52′46″ S, 42°01′07″ W), and at the municipal market of São Pedro in Niterói, Rio de Janeiro, Brazil.

The parasites were released from dissected capsules and fixed with or without compression in alcohol formalin glacial acetic acid fixative (AFA), stained with Langeron alcoholic-acid carmine, dehydrated in an alcohol series, cleared in clove oil and mounted in Canada balsam as permanent slides. Measurements are given in micrometers unless otherwise specified, range values are followed by the mean in parentheses, and the number of specimens is measured in brackets. Light micrographs were taken with a digital camera (Sony MPEGEX, Tokyo, Japan) connected to a light microscope (Zeiss Axioskop, Jena, Germany). Specimens were illustrated with the aid of a camera lucida. The studied specimens are deposited in the *Coleção Helmintológica do Instituto Oswaldo Cruz* (CHIOC), Rio de Janeiro, Brazil.

### 2.2. Genetic and Phylogenetic Analyses

Each capsule collected was separated into two samples: one was morphologically characterized, and the other was separated for genetic analysis. Samples were ground in liquid nitrogen, treated with 10 mg/mL Proteinase K (Invitrogen, Carlsbad, CA, USA) at 56 °C for 12–24 h and the DNA extracted using the DNeasy Plant^®^ MiniKit (QIAGEN, Hilden, Germany), as described by Simões et al. [12]. Negative extraction controls (using tubes without samples) were included. DNA concentrations were estimated at an absorbance of 260 nm in a NanoDrop ND-1000 spectrophotometer (Thermo Fisher Scientific, Wilmington, DE, USA). The samples were submitted to the treatment process utilizing reconstructive polymerization for increasing DNA concentration and volume, following conditions described in the literature [13].

The internal transcribed spacer 2 (ITS2) was used as genetic markers with primers NC13 F (5′-ATCGATGAAGAACGCAGC-3′) and NC2 R (5′-TTAGTTTCTTTTCCTCCGVT-3′), as described by Zhu et al. [14]. Polymerase chain reaction (PCR) was performed with a total volume of 25 µL, containing 2.0 U of Platinum^®^ Taq DNA Polymerase High Fidelity (Invitrogen, Paisley, Scotland), 1Xof High Fidelity Buffer [600 mM Tris-SO_4_ (pH 8.9), 180 mM (NH_4_)_2_SO_4_], 1.5 mM of MgSO_4_, 0.2 mM of dNTPs, 100–200 ng of each primer, 10–50 ng/µL of DNA. The PCR reactions were carried out in a thermocycler Mastercycler Nexus (Eppendorf, Hamburg, Germany), with an initial step of denaturation of 96 °C for 4 min, followed by 40 cycles of 40 s at 96 °C, 40 s at 55 °C and 40 s at 72 °C, and an extension cycle of 5 min at 72 °C. Extraction blank and PCR negative controls were included.

Partial 28S rDNA gene was amplified using the primers 28S F Di (5′ -GTCCGATAGCGAACAAGTACCGT-3′) and 28S R Di (5′-AGCATAGTTCACCATCTTTCGGGTCTCAA-3′) [15]. PCR was conducted with a total volume of 25 µL using 2.5 U of GoTaq^®^ G2 Hot Start Polymerase (Promega, Corporation, Madison, WI, USA), 1X Green GoTaq*^®^* Flexi Buffer, 1.5 mM MgCl_2_, 0.2 mM of dNTPs, 10 µM of each primer, and up to 10 ng/µL of DNA. The thermocycling parameters were performed in a SimpliAmp Thermal Cycler (Life Technologies Corporation, Carlsbad, CA, USA) with an initial denaturation at 96 °C for 4 min, 35 cycles of 96 °C for 40 s, 55 °C for 40 s, 60 °C for 1 min, and a final extension at 72 °C for 5 min.

PCR products of 28S rDNA and ITS2 were analyzed in electrophoresis 1% and 2% agarose gels, respectively, and visualized under UV light after GelRed^®^ (Biotium, Inc., Fremont, CA, USA) staining. The amplified products were purified with the CleanSweep PCR Purification Reagent Kit (Life Technologies Corporation, Carlsbad, CA, USA) and with the EXOSAP-IT Express PCR Product Cleanup Reagent (Life Technologies Corporation, Carlsbad, CA, USA), both following the manufacturer’s protocol. Amplicons were directly sequenced using Big Dye Terminator v. 3.1 Cycle Sequencing Ready Reaction kit (Applied Biosystems) in a 3730 Automated DNA Sequencer (Applied Biosystems) at RPT01A/FIOCRUZ sequencing facility, following the manufacturer’s instructions. Lasergene SeqManTM v.7.0 (DNASTAR, Madison, Wisconsin, USA), Bioedit v.7.0.5 (Department of Microbiology, North Carolina State University, Raleigh, North Carolina), MUSCLE, and GeneDoc v. 2.6.002 were used for sequence editing, aligning and visualization [16,17,18]. Pairwise/BLAST/NCBI searches were performed (http://blast.ncbi.nlm.nih.gov/Blast.cgi, accessed on 2 August 2024) to identify the obtained DNA sequences. Sequences from this study were analyzed and compared with a constructed Didymozoidae dataset of all curated sequences available in GenBank (August 2024) from the literature [10,11,19]. For the treated Didymozoidae dataset, only taxonomically validated sequences were included, supported by publications using specimens curated by specialist taxonomists or referred to institutional biological collections.

Phylogenetic tree constructions were performed by applying Neighbor Joining (NJ), Maximum Likelihood (ML), and Bayesian Inference (BI) methods. NJ and ML phylogenetic analyses were conducted using MEGA version X v.10.1.7 using, for the ITS dataset, Kimura 2-parameter (K2P + G) and Hasegawa–Kishino–Yano (HKY + G) models, respectively, both plus gamma-distributed rate variation among sites, as determined by the best-fit model of DNA substitution based on Bayesian information criteria [20]. In the same way, the 28S rDNA dataset was processed using the K2P + G model, plus gamma distribution for both NJ and ML phylogenetic reconstructions. The statistical support was generated by 500 bootstrap replicates. In addition, BI analysis was subsequently performed (MrBayes), implemented through TOPALi v. 2.5 [21] and on the BEAST 2.5 platform [22], using K2P + G, the best-fit model selected based on the Bayesian information criterion.

## 3. Results

### 3.1. Morphological Taxonomy

**Figure 1:**
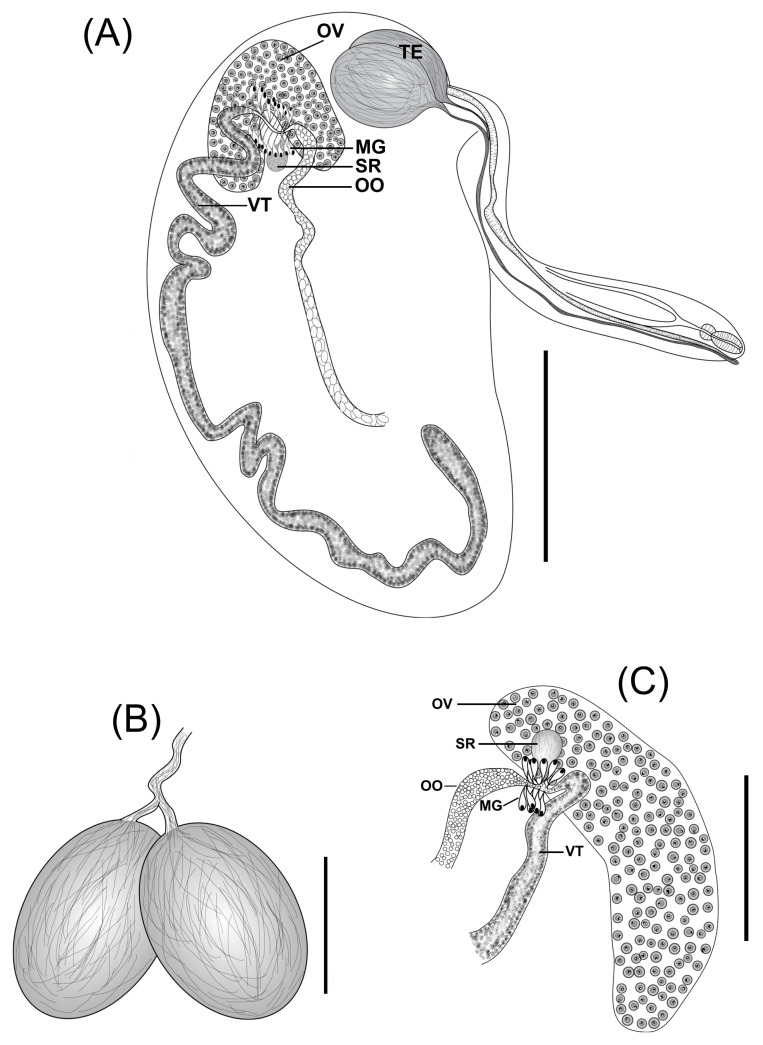
*Platodidymocystis yamagutii* n. gen., n. sp. (**A**) Whole mount (Holotype), (**B**) detail of testes (**C**) detail of a genital junction. Testes (TE), ovary (OV), vitellarium (VT), oötype (OO), Mehlis’ gland (MG), and seminal receptacle. (SR) Scale bars: (**A**) 400 μm (**B**) 90 μm (**C**) 140 μm.

**Figure 2:**
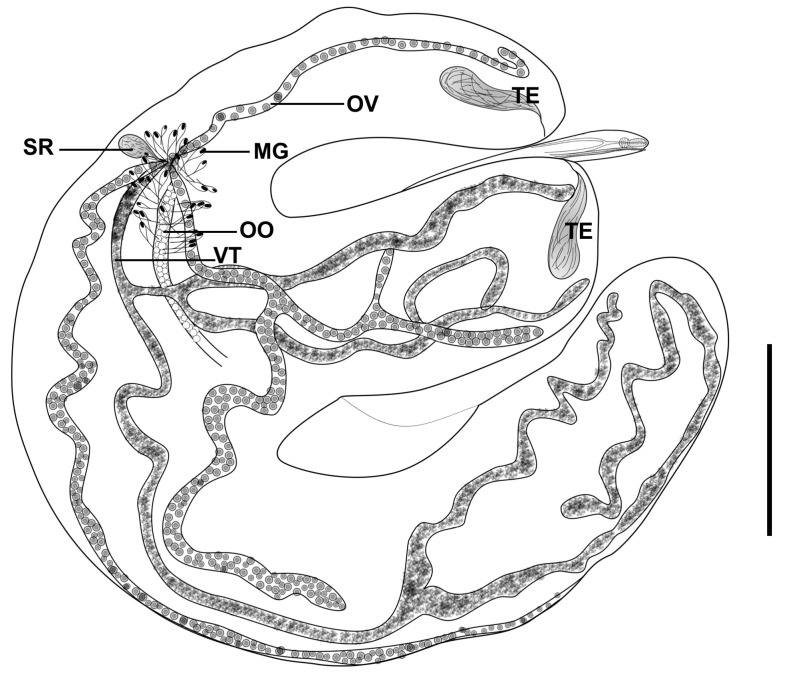
*Didymosulcus philobranchiarca*. Whole mount. Testes (TE), ovary (OV), vitellarium (VT), oötype (OO), Mehlis’ gland (MG), and seminal receptacle (SR). Scale bars: 0.80 mm.

**Figure 3:**
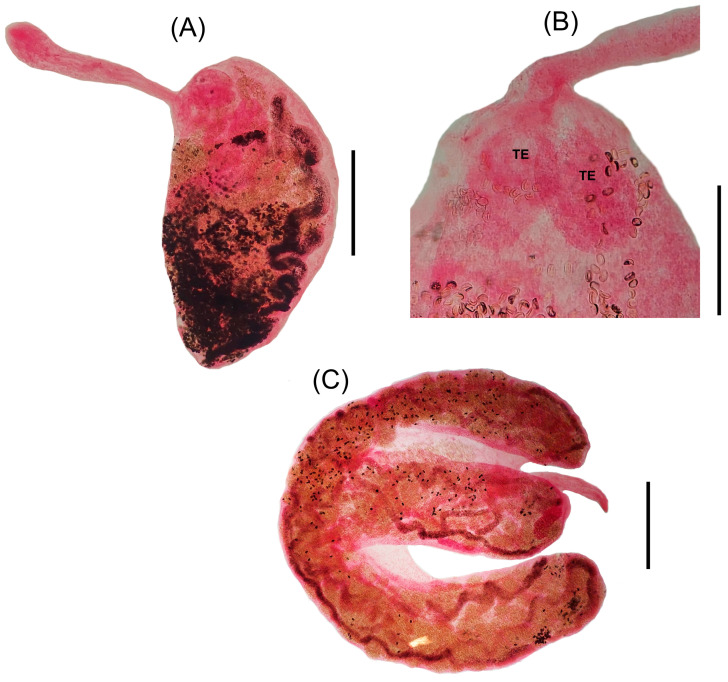
Photomicrographs of *Platodidymocystis yamagutii* n. gen., n. sp. and *Didymosulcus philobranchiarca* stained with Langeron alcoholic-acid carmine. (**A**) *Platodidymocystis yamagutii* n. gen., n. sp., whole specimen (**B**) Detail showing the rounded testes of *Platodidymocystis yamagutii* n. gen., n. sp. (**C**) *Didymosulcus philobranchiarca*, whole specimen. Scale bars: (**A**) 300 µm (**B**) 140 µm (**C**) 800 µm.

From the *T. obesus* examined, five didymozoid species were found: *Didymocystis neothunni* (Yamaguti, 1970) Pozdnyakov, 1996, *Didymosulcus philobranchiarca* (Yamaguti, 1970) Pozdnyakov, 1990, *Koellikerioides internogastricus* Yamaguti, 1970, *Platocystis vivipara* (Yamaguti, 1970) Pozdnyakov, 1987, *Platodidymocystis yamagutii* n. gen., n. sp. (Figure 1, Figure 2 and Figure 3).

**Description**

Class Trematoda Rudolphi, 1808

Subclass Digenea Carus, 1863

Order Plagiorchiida La Rue, 1957

Superfamily Hemiuroidea Looss, 1899

Family Didymozoidae Monticelli, 1888

Subfamily Didymozoinae Monticelli, 1888

### 3.2. Platodidymocystis n. gen.

Diagnosis: A globular capsule containing two hermaphroditic individuals. Tegument thin. Body divided into two distinct regions: anterior region slender, very long, reaching more than half the size of the posterior region. Posterior region flattened, semicircular. Oral sucker subterminal. Pharynx present. Esophagus narrow. Ventral sucker absent. Two testes predominantly rounded, rarely oval. Genital pore well developed, opening ventrolateral to oral sucker. Vitellarium tubular, single. Ovary short and wide, approximately oval in shape, sometimes recurved, not branched. Oötype surrounded by weakly developed Mehlis’ gland. Seminal receptacle present. Uterus occupying all available space of the posterior region. Egg reservoir absent. Excretory system not observed. Encysted under the dorsal skin and the caudal fin of Scombridae. Southwestern Atlantic Ocean.

Type-species: *Platodidymocystis yamagutii* n. gen., n. sp.

Etymology: the genus is named according to the similarity of the morphological characteristics of the new genus to the already known genera *Platocystis* and *Didymocystis*.

#### 3.2.1. *Platodidymocystis yamagutii* n. gen., n. sp. (Figure 1A–C and Figure 3A,B)

Type host: *Thunnus obesus* (Lowe, 1839) (Scombriformes: Scombridae)

Type locality: Off Cabo Frio (22°52′46″ S, 42°01′07″ W), Rio de Janeiro, Brazil

Site of infection: Dorsal skin and the caudal fin

Total number of parasites: 58

Prevalence: 2.1% (1 parasitized out of 47 examined)

Intensity: 58

Specimens deposited: Holotype CHIOC 40453 a; Paratypes CHIOC 40453 b-m.

Etymology: The specific name is in honor of Prof. Satyu Yamaguti, who made significant contributions to the knowledge of Didymozoidae through publications, which are still in use.

Fifty-eight specimens of *Platodidymocystis yamagutii* n. gen., n. sp. were observed as yellowish capsules rounded, flattened, under the dorsal skin and caudal fin containing two hermaphroditic individuals similar in form and size in one out of 47 *T. obesus* examined.

Description (based on 48 specimens): Body divided into two distinct regions. Anterior region slender, very long (Figure 1A; 3A), reaching more than half the size of the posterior region, 350–950 (634) long, with a maximum width of 80–130 (105) [*n* = 44] in the esophageal region, connected to the anterior region of the flat margin of the posterior region. Posterior region flattened semicircular (Figure 1A; 3A) 410–1200 (831) long by 290–720 (493) wide [*n* = 45]. Oral sucker subterminal, piriform, muscular 42–62 (56) long by 25–37 (33) wide [*n* = 47]. Pharynx 17–30 (22) long by 20–27 (24) wide [*n* = 47]. Esophagus narrow 75–135 (109) [*n* = 26] long. Caecum clearly visible in the anterior region but not traceable in the posterior region (Figure 1A). Testes predominantly rounded (Figure 1A) to oval (Figure 1B and Figure 3B), paired, sometimes juxtaposed, closed to flat margin near the anterior end of the posterior region, 83–163 (144) long by 60–157 (97) wide [*n* = 41]; vas deferens well visible in the anterior region running straightforward along with metraterm. Genital pore well developed, opening ventrolateral to oral sucker (Figure 1A). Genital junction located in the anterior part of the posterior region (Figure 1A,C). Ovary consists of a short and wide structure, approximately oval, sometimes recurved, not branched (Figure 1A,C), situated in the anterior part of the posterior region 75–165 (120) [*n* = 4] wide 275–450 (323) long. Mehlis’ gland surrounds the uterine oötype with few dense gland cells. Seminal receptacle rarely visible. Vitellarium single, tubular, 25–65 (45) wide [*n* = 44], extending from the initial portion of the posterior region, presenting conspicuous inward loops along the whole convex margin (Figure 1A Uterus occupying all available space of posterior region, not forming egg reservoir (Figure 3A). Eggs embryonated 13–16 (14) long by 8–11 (10) wide [*n* = 280].

Remarks: The new species, herein proposed, was found in capsules containing two hermaphroditic individuals, similar in form and size, with a body divided into two distinct regions, one anterior elongate and narrow and one posterior enlarged. Considering these characteristics, the new genus was allocated to Didymozoinae, differing from all known members of the subfamily mainly by the morphology of the testes and ovary (rounded testes and ovary short and wide). *Platodidymocystis* n. gen. is more closely related to *Platocystis* mainly by the arrangement of a single ovary and vitellarium non-branched and to *Didymocystis* by the shape of the body.

*Platodidymocystis yamagutii* n. gen., n. sp. differs from *Platocystis alalongae* mainly by the ovary (a short and wide structure, approximately oval, sometimes recurved, not branched in the new species vs. tubular ovary divided into two main branches, each of which is subdivided in *P. alalongae*); by the vitellarium (tubular, presenting conspicuous inward loops along the whole convex margin in *P. yamagutii* n. gen., n. sp. vs. shorter anterior and very long posterior tubules coiled along the whole convex margin of the posterior region in *P. alalongae*); by the testes (rounded to oval, paired, sometimes juxtaposed, closed to flat margin near anterior end of posterior region in the new species vs. convoluted vermiform testes lie in the posterior region near the base of the anterior region in *P. alalongae*). The new species differs from *P. alalongae* and from *Platocystis meridionalis* Pozdnyakov, 1987 by the posterior region comparatively smaller (4.7–6.0 mm by 2.35–3.6 mm in *P. alalongae*, 4.91–5.54 mm by 2.58 mm in *P. meridionalis* vs. 410–1200 by 290–720 in *P. yamagutii* n. gen., n. sp.). The new species is most similar to *P. vivipara* and *P. viviparoides*, mainly by presenting a single ovary and vitellarium, not branched, by parasitizing the same host species and a congener (*T. albacares*), and by the specific habitat but differs principally by the shape of ovary that is tubular, long, extending windingly along longitudinal of posterior region and by the testes long and sausage-shaped in the two previously known species. *Platodidymocystis yamagutii* n. gen., n. sp. is also related to *Didymocystis pectoralis* (Yamaguti, 1970) Pozdnyakov, 1990, described at base of inner surface of pectoral fin of *T. obesus*, by the same site of infection and by the ovary and vitellarium not branched. Considering the shape of testes, the new genus and species reminds us of *Didymosulcus rotunditestis* (Nikolaeva & Dubina, 1985) Pozdnyakov, 1990 and *Allopseudocolocyntotrema claviforme* Yamaguti, 1970, but differs in the shape of the ovary, vitellarium, and site of infection.

#### 3.2.2. *Didymosulcus philobranchiarca* (Yamaguti, 1970) Pozdnyakov, 1990 (Figure 2 and Figure 3C)

Host: *Thunnus obesus* (Lowe, 1839) (Scombriformes: Scombridae)

Locality: Off Cabo Frio (22°52′46″ S, 42°01′07″ W), Rio de Janeiro, Brazil.

Site of infection: encapsulated in pairs in hard denticle palate and gill arches

Total number of parasites: 34

Prevalence: 8.5% (4 parasitized out of 47 examined)

Specimens deposited: Voucher CHIOC (waiting approval of paper)

New data (based on 10 specimens): rounded capsules of bright yellow containing two hermaphroditic individuals, similar in shape and size. Body divided into two distinct regions (Figure 2 and Figure 3C). Anterior region tapered 0.67–1.47 (1.04) mm × 0.15–0.2 (0.17) mm connected in the anterior part of the posterior region of the body between two lobes. Posterior region comma-shaped, measuring 3.05–5.15 (4.3) mm × 1.1–2.15 (1.59) mm, presenting a pronounced longitudinal groove that divides the anterior portion into two distinct lobes, the formation of lobes starting approximately in the middle of the body. Mouth terminal. Oral sucker pyriform 45–50 (49) by 35–37 (36) directly followed by a subcylindrical pharynx 35–47 (41) by 30–47 (44). Esophagus simple, 400–750 (510) [*n* = 8] long. Ceca narrow in the anterior region, expanding in the posterior region (Figure 2). Testes tubular, 650–1130 (931) by 130–230 (175) [*n* = 9] located in the anterior portion of the lobes of the posterior region. Vas deferens running alongside the metraterm in the anterior region of the body. Genital pore ventrolateral to the oral sucker. Ovary tubular, divided into four branches, most of which go to the anterior lobe region, and one goes to the final part of the posterior region, 60–120 (82) [*n* = 9] wide. Small seminal receptacle seen in a single specimen (Figure 2). Mehlis’ gland surrounds the uterine oötype with few dense gland cells. Vitellarium with three main branches that bifurcates originating eight terminal branches 60–80 (68) wide (Figure 2). Uterus sinuous, occupying the entire space in the posterior region of the body (Figure 3C). Egg reservoir occupies a large convex part of the posterior region of the body. Eggs operculated, 15–17 (16) by 9,10 (10) [*n* = 100].

Remarks: *Didymosulcus philobranchiarca* was described parasitizing the gill arches of *T. albacares* (= *Neothunnus macropterus*) and *T. obesus* (= *Parathunnus sibi*) from Hawaii, Pacific Ocean [23], and posteriorly transferred to *Didymosulcus* by Pozdnyakov [24]. This species was also reported in *T. obesus* and *Thunnus alalunga* from the Indian Ocean [25]; and in *T. albacares*, *T. obesus*, and *T. atlanticus* from the Atlantic Ocean [26]. The morphology of the specimens studied in the present study was in accordance with the original description, differing only in some measurements, with the species collected in the Atlantic, including the specimens studied by Justo et al. [26], being comparatively larger than those collected in the Pacific by Yamaguti [23] and Nikolaeva and Dubina [25].

### 3.3. Genetic and Phylogenetic Analyses of Didymozoidae Species

**Figure 4:**
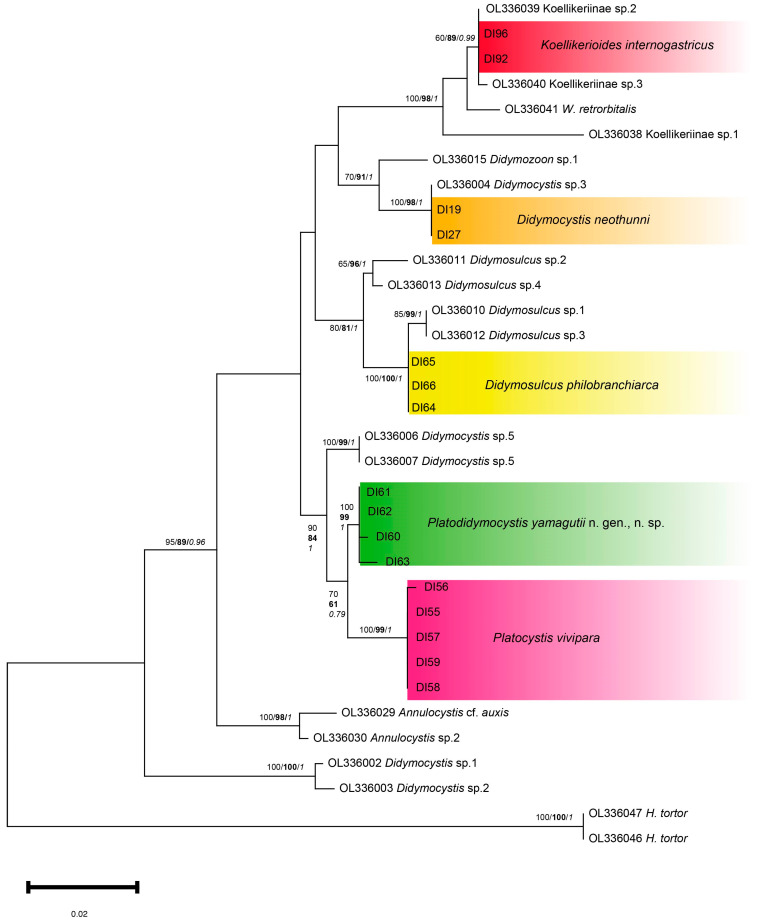
Phylogenetic tree based on partial 28S rDNA of Didymozoidae collected from *T. obesus* from Brazilian Atlantic coast using ML method, K2P plus gamma distribution and constructed on MEGA v. X. Sub-clusters of the newly generated sequences from Didymozoidae species from the present study are presented in color-graded shades. Nodal numbers at branches are statistical support based on ML (regular), NJ (bold), and BI (italic) methods. Only statistical values >60% and >0.6 are shown. *Helicodidymozoon tortor* species was used as the outgroup.

Samples from the five Didymozoidae species, *Koellikerioides internogastricus* (*n* = 6), *Didymocystis neothunni* (*n* = 3), *Didymosulcus philobranchiarca* (*n* = 3), *Platocystis vivipara* (*n* = 7), and *Platodidymocystis yamagutii* n. gen., n. sp. (*n* = 5) were submitted to genetic analyses using 28S rDNA and ITS2 markers. The 28S rDNA analysis showed 16 sequences of 500–600 bp from *K. internogastricus* (*n* = 2; DI92 and DI96), *D. neothunni* (*n* = 2; DI19 and DI27), *D. philobranchiarca* (*n* = 3; DI64–DI66), *P. vivipara* (*n* = 5; DI55–DI59) and *P. yamagutii* n. gen., n. sp. (*n* = 4; DI60–DI63). The 28S rDNA phylogenies based on the three methods were similar in topology. In general, all the sequences generated in the present study were grouped in genus-specific clusters (Figure 4).

The two 28S rDNA sequences obtained from *K. internogastricus* specimens were identical and formed a species-specific cluster together with sequences deposited as Koellikeriinae sp. 2 and Koellikeriinae sp. 3 [10], with strongly (BI = 0.99), moderate (NJ = 89%) to weakly (ML = 60%) supported statistical values (Figure 4). The cluster containing the new *K. internogastricus* sequences is placed in a big group that comprehended other Koellikeriinae subfamily sequences, *Wedlia retrorbitalis* and Koellikeriinae sp. 1, with high bootstrap support (ML = 100%; NJ = 98%; BI = 1) (Figure 4).

*Didymocystis neothunni* new sequences clustered, with full (ML = 100%; BI = 1) to strong support (NJ = 98%) with a unique sequence named *Didymocystis* sp. 3, by Louvard et al. [10] (Figure 4). Other *Didymocystis* clusters are observed in the 28S rDNA phylogenetic trees (Figure 4). A second cluster with statistical values fully (ML = 100%; BI = 1) to strongly support (NJ = 99%), shaped with *Didymocystis* sp. 5 sequences and a third one containing *Didymocystis* sp. 1 and *Didymocystis* sp. 2 (ML/NJ = 100%; BI = 1), all sequences by Louvard et al. [10] (Figure 4).

*Didymosulcus philobranchiarca* 28S rDNA sequences generated in this study were identical (DI64–DI66) and grouped with maximum statistical support (ML/NJ = 100%; BI = 1) with a clade of *Didymosulcus* sp. 1 and *Didymosulcus* sp. 3, named by Louvard et al. [10] (Figure 4). This cluster is placed in a monophyletic group only with *Didymosulcus* spp. sequences, nodal fully (BI = 1) to moderately supported (ML = 80%; NJ = 81%), which seems to be a genus-specific cluster (Figure 4).

*Platodidymocystis yamagutii* n. gen., n. sp. 28S rDNA sequences (DI60–DI63) were grouped in a monophyletic cluster with full (ML = 100%; BI = 1) to strong statistical support (NJ = 99%) and placed close to the *Platocystis vivipara* cluster from this study with moderate (ML = 70%; BI = 0.79) to weak support (NJ = 61%). *Platodidymocystis yamagutii* n. gen., n. sp. and *P. vivipara* clusters form a group next related to a cluster of *Didymocystis* sp. 5 sequences, with nodal fully (BI = 1), strongly (ML = 90%) to moderately supported (NJ = 84) (Figure 4).

*Platocystis vivipara* 28S rDNA sequences from this study (DI55–DI59) produced a well-defined monophyletic cluster with statistical values fully (ML = 100%; BI = 1) to strongly support (NJ = 99%) closely related to the new sequence obtained from *Platodidymocystis yamagutii* n. gen., n. sp. (Figure 4), as mentioned above.

The ITS2 analysis showed that 23 sequences of 400-500 bp in length were obtained from *K. internogastricus* (*n* = 6; DI01–DI02, DI06, DI08, DI92, and DI96), *D. neothunni* (*n* = 3; DI07, DI19 and DI27), *D. philobranchiarca* (*n* = 3; DI64–DI66), *P. vivipara* (*n* = 7; DI10, DI12 and DI55–DI59) and *P. yamagutii* n. gen., n. sp. (*n* = 3; DI13 and DI60–DI63).

**Figure 5:**
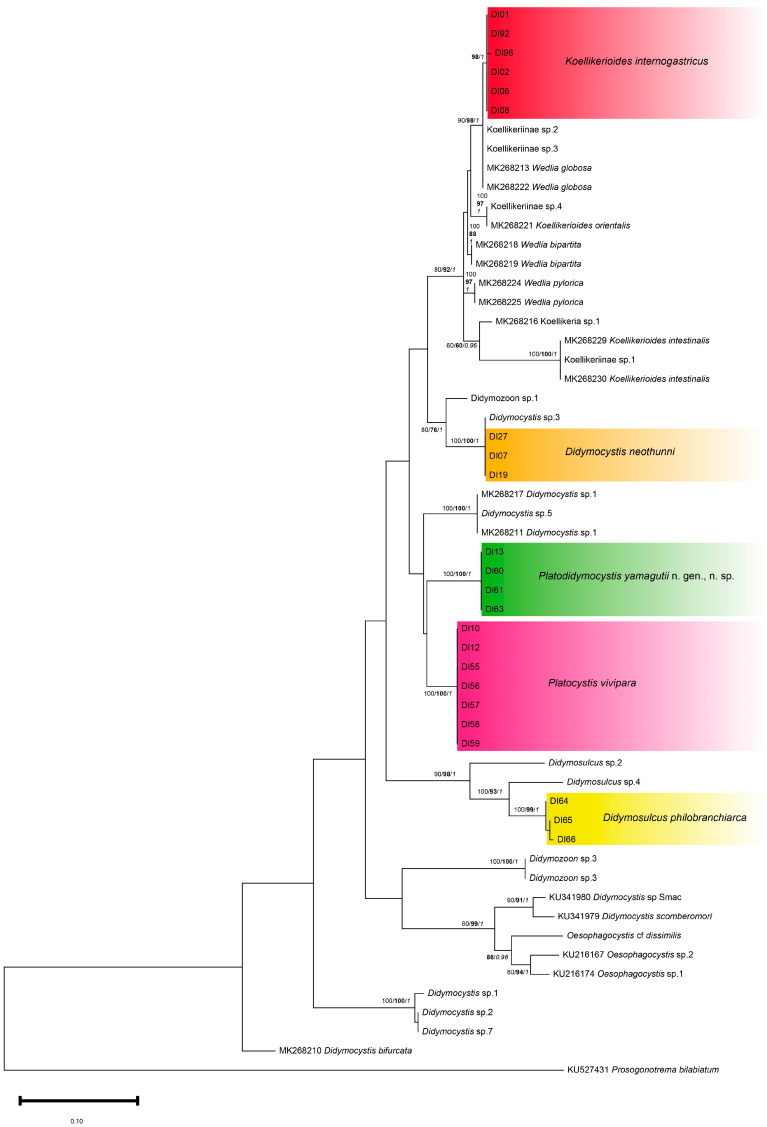
Phylogenetic tree based on partial ITS2 region of Didymozoidae collected from *T. obesus* from Brazilian Atlantic coast using ML method, HKY + G distribution and constructed on MEGA v. X. Sub-clusters of the newly generated sequences from Didymozoidae species from the present study are presented in color-graded shades. Nodal numbers at branches are statistical support based on ML (regular), NJ (bold), and BI (italic) methods. Only statistical values >60% and >0.6 are shown. *Prosogonotrema bilabiatum* was used as the outgroup.

The ITS2 phylogenetic trees based on the three methods were similar in topology, grouping the samples from the present study in the same clusters with high to moderated bootstrap values (Figure 5). The six ITS2 sequences obtained in the present study from *K. internogastricus* specimens formed a species-specific discrete cluster with high support values for NJ and BI methods but not for ML reconstruction (ML < 60%; NJ = 98%; BI = 1) (Figure 5). They are closed to Koellikeriinae sp. 1, Koellikeriinae sp. 3, and *Wedlia globosa* Ishii, 1935 sequences (ML = 90%; NJ 98%; BI = 1). The cluster containing the new *K. internogastricus* sequences is placed in a big group that comprehended some species-specific groups from Koellikeriinae (ML = 80%; NJ = 92%; BI = 1), such as *K. intestinalis*, *K. orientalis*, *W. bipartita*, and *W. pylorica* (Figure 5). Interestingly, a well-defined topology and maximum to strong statistical values of *K. intestinalis* (ML/NJ = 100%; BI = 1) and *K. orientalis* (ML = 100%; NJ = 97%; BI = 1) clusters with Koellikeriinae sp. 1 and Koellikeriinae sp. 4, respectively, were observed (Figure 5). The are only three ITS2 nucleotide sequences available corresponding to *Koellikerioides* species:* K. intestinalis*, *K. orientalis*, and *K. apicalis*. The last one was omitted from our ITS2 analysis due to its short length, 163 bp or 189 bp [FJ628683-6 and AB725622] [15].

*Didymocystis neothunni* new sequences clustered together and with a unique sequence named *Didymocystis* sp. 3, with maximum statistical support (ML/NJ = 100%; BI = 1) (Figure 5). Other three *Didymocystis* clusters are observed in the ITS2 phylogenetic tree demonstrating the characteristic polyphyly of the genus (Figure 4).

*Platodidymocystis yamagutii* n. gen., n. sp. sequences (DI13 and DI60-DI63) generated were placed isolated in the phylogeny, as expected, with maximum support values (ML/NJ = 100%; BI = 1). The closest group was the *Platocystis vivipara* cluster from this study. *Platodidymocystis yamagutii* n. gen., n. sp. and *P. vivipara* clusters form a group next related to a *Didymocystis* spp. cluster, also with maximum support values (ML/NJ = 100%; BI = 1), but contained reference sequences that were not morphologically identified at the species level (Figure 4).

*Platocystis vivipara* ITS2 sequences from this study were identical and produced a cluster, closely related to the *Platodidymocystis yamagutii* n. gen., n. sp. clade, as mentioned above. There are no reference sequences from the *Platocystis* genus with a suitable length available for the present ITS2 phylogenetic analysis, only the shortened from *P. alalongae* [FJ628687-9].

*Didymosulcus philobranchiarca* sequences generated in this study were grouped, with nodal fully (ML = 100%; BI = 1) or strongly supported (NJ = 99%). The *D. philobranchiarca* clade is contained in a major clade with only *Didymosulcus* spp. sequences in monophyly, fully (BI = 1) or strongly supported (ML = 90%; NJ = 98%), that seems to be a genus-specific cluster for *Didymosulcus* spp. However, those reference sequences were not morphologically identified at the species level and named *Didymosulcus* sp. 2 and *Didymosulcus* sp. 4 (Figure 4). Four *Didymosulcus* species identified have ITS2 sequences available, *D. wedli* [AB725625, FJ628703], *D. irregularis* [FJ628684], *D. palati* [FJ628690, 702, 716] and *D. spirocauda* [FJ628677, FJ628679], but absent from our phylogenetic analysis due to their shortened length, 188 and 163 bp [15].

## 4. Discussion

In South America, many didymozoid species have been reported from marine [16,17,18,19,20,21,22,23,24,25,26,27] and freshwater fishes [5]. Studies on parasites of *T. obesus* have shown a great diversity of species of didymozoid [26,27,28,29]. The Didymozoidae contains six subfamilies including Didymozoinae, which comprises nineteen valid genera [4]. In this study, *Platodidymocystis* n. gen. is proposed to accommodate *P. yamagutii* n. gen. n sp. described from *T. obesus*, differing from all other members of Didymozoinae mainly by the morphology of the testes and ovary (rounded testes and ovary short and wide). Considering morphology, *Platodidymocystis* n. gen. is more closely related to *Platocystis*, mainly in the arrangement of a single ovary and vitellarium non-branched and to *Didymocystis* by the shape of the body.

The 28S rDNA and ITS2 sequences obtained in the present study agree with the morphological characterization that recognized the species *D. neothunni*, *K. internogastricus*, *P. vivipara*, *D. philobranchiarca*, and *Platodidymocystis yamagutii* n. gen. n. sp., parasitizing *T. obesus* from the littoral of Rio de Janeiro State, Brazil. The morphology of *K. internogastricus*, *D. neothunni*, *P. vivipara* was given in previous studies by Justo et al. [28], Justo and Kohn [29], and Moreira-Silva et al. [27]. The molecular taxonomy demonstrated *Platodidymocystis yamagutii* n. gen. n. sp. cluster located isolated in the phylogeny, closely related to *Platocystis vivipara* and *Didymocystis* spp. clusters, in agreement with the morphology. Regarding the hosts, the 28S rDNA and ITS2 phylogenies showed that the *Didymocystis* spp. cluster contained sequences from Louvard et al. [10] named *Didymocystis* sp. 5, which also infected *T. obesus*, and *Didymocystis* sp. 1 YFT, and *Didymocystis* sp. 1 BET-A (MK268211 and 17), collected from *T. albacares* and *T. obesus*, respectively [11] (Figure 4), from Australia, Indonesia, and Solomon Island coasts, respectively. All the samples that grouped to *Platodidymocystis yamagutii* n. gen. n. sp., from this study and references are from *Thunnus* spp. Regarding the site of infection, *Platodidymocystis yamagutii* n. gen. n. sp. was found in dorsal skin and caudal fin, the *P. vivipara* was collected from the tegument of the dorsal region of the body [27], and the *Didymocystis* spp. samples, under the skin of the inner face of the operculum and gills [10,11]. Mladineo et al. [15] performed a phylogenetic analysis of Didymozoidae parasites of two scombrid fishes: *Thunnus orientalis* (Temminck and Schlegel, 1844) in the Pacific, and *Thunnus thynnus* (Linnaeus, 1758) in the Atlantic, using part of 28S rDNA, ITS2, and the mitochondrial cytochrome oxidase 1 gene (*cox*1). The data obtained by these authors suggested that didymozoids belong to a group of parasites in which habitat is the leading force in shaping the phylogenetic relationships. During their evolution, didymozoids spread and inhabit different sites of infection, colonizing both the exterior and strict interior niches, undergoing evolutive changes in the site of infection, as well as in the morphology of the posterior part of the body [15]. *Platodidymocystis yamagutii,* n. gen., n. sp. inhabits the same site of infection as *Didymocystis pectoralis*, a feature of relevance to the taxonomy of the group, considering that the species of Didymozoidae are pointed as site-specific [23]. However, Louvard et al. [10] argue about whether the infection site is a relevant characteristic of taxonomic importance in the definition of some Didymozoidae species that seem to be highly site-specific. On the other side, the authors highlighted that the site specificity character is an untested hypothesis of species definition and that, for example, the only difference between the species of *Koellikerioides intestinalis* Yamaguti, 1970 and *Koellikerioides apicalis* Yamaguti, 1970 is their infection site. While the value of this character remains under debate, taxonomic integrative studies, that generate morphological and genetic data of didymozoid species, are necessary for more robust species definition and to test the force of these characters in the evolutionary history of each taxon. The genetic data and molecular phylogenies obtained in the present study of *P. yamagutii* n. gen., n. sp., using both the 28S rDNA gene and ITS2 genetic markers, also corroborate the morphological data, considering that the new genus is closely related to *Platocystis* sharing morphological features.

Regarding the *P. vivipara* sequences obtained, no *Platocystis* spp. reference sequences, except for the short *P. alalongae* sequences, are available for the ITS2 analysis. Herein, we presented the first genetic information from *P. vivipara* from taxonomically curated samples of species (deposited specimens: CHIOC n° 40,089, 40,090 a-e; Helminthological collection of the Oswaldo Cruz Institute, CHIOC by Moreira-Silva et al. [27].

In both 28S rDNA and ITS2 *Didymocystis neothunni* phylogenies, the cluster that contains the new sequences obtained from the parasite found encapsulated in the membrane that covers the ventral side of the tongue of *T. obesus* in the present study is grouped in a well-supported monophyletic clade and with a sequence named *Didymocystis* sp. 3, isolated from the seam of the lip of a *T. albacares* from the Australian coast. Considering the high statistical values that support the monophyletic clade, and the morphological curated characterization by Justo et al. [29] (deposited specimens: CHIOC n° 37,128 a–d, 37,129), it is possible to suggest that probably *Didymocystis* sp. 3 by Louvard et al. [10] could be the species *D. neothunni*. The result suggests a large geographical distribution of the *D. neothunni* driven by their *Thunnus* spp. hosts.

More distinct *Didymocystis* spp. clusters are observed in both 28S rDNA and ITS2 phylogenetic trees, demonstrating the polyphyletic character of this genus, as previously mentioned by Louvard et al. [10].

The genetic analysis of *K. internogastricus* from the present study, found parasitizing *T. obesus* from the Brazilian Atlantic coast, exhibited a 28S rDNA conserved cluster including the Koellikeriinae sp. 2 sequence, and a more polymorphic ITS2 reconstruction showed a discrete species-specific cluster, with low to high statistical support (Figure 5), near to sequences from *W. globosa* and Koellikeriinae sp. 2 and Koellikeriinae sp. 3. These Koellikeriinae reference sequences were collected from *T. albacares* and *T. obesus* hosts in diverse sites of infections [10]. The *Koellikerioides internogastricus* ITS2 clade from this study is included in a large group that seems a Koellikeriinae clade with *Koellikerioides* and *Wedlia* species-specific groups. These well-supported species-specific clusters allow us to propose the identification of sequences named Koellikeriinae sp. 1 and Koellikeriinae sp. 4 as belonging, in fact, to *K. intestinalis* and *K. orientalis*, respectively. The present study makes available the first sequences of *K. internogastricus* parasitizing *T. obesus* from the Brazilian Atlantic coast from curated specimens following Justo et al. [28] (voucher specimens deposited CHIOC n: 37,004, 37,005, 37,006, 37,007, 37,008, 37,009, 37,010, 37,011).

*Didymosulcus philobranchiarca* was characterized herein by morphological and molecular analyses. The new *D. philobranchiarca* sequences showed a well-defined species-specific cluster, as indicated by the ITS2 phylogenetic reconstructions. Using both genetic markers, the parasites, collected from the hard denticle palate and gill arches of *T. obesus*, were grouped in a strongly supported *Didymosulcus* genus-specific clade. The *D. philobranchiarca* 28S rDNA sequences were closer to a cluster containing *Didymosulcus* sp. 1 and *Didymosulcus* sp. 3 sequences, from *T. albacares* and *T. obesus* hosts, respectively, and near a clade of *Didymosulcus* sp. 2 and *Didymosulcus* sp. 4 sequences, also from *T. albacares* and *T. obesus* hosts, respectively, all from the Australian coast [10]. The ITS2 phylogenetic reconstruction revealed that the *D. philobranchiarca* clade was nearer to *Didymosulcus* sp. 4 than *Didymosulcus* sp. 2 sequences, from *T. obesus* and *T. albacares* hosts, respectively. The maps of the native range of both species of tunas show that they occur in sympatry. The sympatric and the highly migratory behaviors could explain the sharing of *Didymosulcus* spp. parasites. Regarding the infection sites, the *Didymosulcus* spp. used as reference sequences in the present study were also collected from the palate and gills, except by *Didymosulcus* sp. 3, which was found in retrorbital adipose tissue.

## 5. Conclusions

Integrative taxonomic studies of Didymozoidae are rare, and genetic data lack accurate species identification. This study used an integrative taxonomy based on morphological and genetic data to identify didymozoids infecting tunas. Both approaches agree with the proposition of a new genus and species and the identification of species already known. The new genus and species, *Platodidymocystis yamagutii* n. gen., n. sp., revealed a molecular phylogeny of a unique cluster in monophyly, most closely related to *Platocystis* spp., followed by *Didymocystis* spp., in agreement with the morphology. In addition to the new genus and species described in the present study, *Didymosulcus philobranchiarca* was characterized by integrative taxonomy, and three more species were analyzed via molecular taxonomy, *Koellikerioides internogastricus*, *Didymocystis neothunni*, and *Platocystis vivipara*, corroborating with the morphological taxonomy, contributing with new genetic data. This is the first integrative taxonomic study on Didymozoidae from the Southwest Atlantic coast, providing the first 28S rDNA and ITS2 genetic data from five morphological curated species. The results of the present work support that integrative studies are necessary to elucidate taxonomic problems, especially in cases where the taxonomy is confusing, as in the family Didymozoidae, contributing to the knowledge about their complex phylogeny, biology, geographical, host ranges and the host–parasite relationships.

## Data Availability

All sequences generated from this study have been submitted to the GenBank database.
